# Advanced MMC-Based Hydrostatic Bearings for Enhanced Linear Motion in Ultraprecision and Micromachining Applications

**DOI:** 10.3390/mi16050499

**Published:** 2025-04-24

**Authors:** Ali Khaghani, Atanas Ivanov, Mina Mortazavi

**Affiliations:** 1 Department of Mechanical and Aerospace Engineering (MAE), Brunel University London, Uxbridge UB8 3PH, UK; atanas.ivanov@brunel.ac.uk; 2 School of Computer Science and Technology, University of Bedfordshire, University Square, Luton LU1 3JU, UK; mina.mortazavi@beds.ac.uk

**Keywords:** ultraprecision machining, micromachining, micropositioning, linear direct actuator, dynamics

## Abstract

This study investigates the impact of material selection on the performance of linear slideways in ultraprecision machines used for freeform surface machining. The primary objective is to address challenges related to load-bearing capacity and limited bandwidth in slow tool servo (STS) techniques. Multi-body dynamic (MBD) simulations are conducted to evaluate the performance of two materials, alloy steel and metal matrix composite (MMC), within the linear slideway system. Key performance parameters, including acceleration, velocity, and displacement, are analyzed to compare the two materials. The findings reveal that MMC outperforms alloy steel in acceleration, velocity, and displacement, demonstrating faster response times and greater linear displacement, which enhances the capabilities of STS-based ultraprecision machining. This study highlights the potential of utilizing lightweight materials, such as MMC, to optimize the performance and efficiency of linear slideways in precision engineering applications.

## 1. Introduction

Hydrostatic and aerostatic bearings are commonly employed in micromachining and ultraprecision machining systems to support linear slideways. These slideways are often integrated with slow tool servo (STS) and fast tool servo (FTS) techniques. The application of the STS technique for machining freeform surfaces can pose challenges regarding motion performance and accuracy [1]. Achieving sub-micron level accuracy is particularly difficult and requires a robust slideway design capable of compensating for external influences such as thermal expansion, vibrations, and friction [2]. The use of high-pressure liquids in hydrostatic bearings enables the external generation of load-carrying capacity [3,4]. The high-pressure liquid, typically oil, used in hydrostatic bearings introduces several viscous damping effects. The bearing clearance typically ranges between 10 and 100 μm. Due to precise engineering requirements, only a limited number of manufacturers produce hydrostatic bearing components. In ultraprecision machine design, hydrostatic bearings are often custom-made to meet specific customer requirements, with no standardized oil pressure or resource specifications. As such, the design of hydrostatic bearings is largely dictated by the required load capacity [5].

Hydrostatic bearings that use incompressible liquids under high pressure offer several benefits, including high damping, stiffness, pressure distribution, and load-carrying capacity [6]. Even with imperfect surfaces, the error motion in hydrostatic bearings typically remains below 100 nm [7]. However, their performance in high-speed applications is limited due to dynamic friction coefficients. Achieving sub-micron accuracy necessitates minimizing power losses and thermal effects. The STS technique depends on hydrostatic bearings for their stiffness, damping, and linear accuracy [8]. Nonetheless, the limitations in speed and thermal effects must be carefully considered. This study aims to assess the motion accuracy of hydrostatic bearings in ultraprecision machining systems by evaluating straightness. Additionally, it investigates the potential of using lightweight metal matrix composites (MMCs) to improve motion robustness and dynamics in hydrostatic bearing slideways for STS mode. The structural integrity of hydrostatic bearings incorporating MMC materials will also be examined. The metal matrix composite (MMC) material chosen for this study consists of an aluminum matrix reinforced with silicon carbide (SiC) particles. This specific composition was selected due to its superior mechanical properties compared with traditional materials like alloy steel. The aluminum matrix offers a low density, contributing to a reduced overall weight, while the silicon carbide reinforcement enhances the material’s stiffness and wear resistance [9,10,11]. These characteristics are particularly beneficial in ultraprecision machining, where high acceleration and velocity are required, and where maintaining a low weight without compromising structural integrity is crucial. Furthermore, the high thermal conductivity of MMCs helps in mitigating temperature-induced deformations, which is a common challenge in ultraprecision machining. The combination of these properties makes the selected MMC an ideal candidate for the design of linear slideways in ultraprecision machines, offering significant advantages in terms of dynamic behaviour, response times, and overall system efficiency [12].

The choice of Al-SiC over other MMC compositions, such as TiC-reinforced or other Al-based composites, was driven by the balance it offers between mechanical performance, thermal properties, and cost-effectiveness. While TiC composites provide superior hardness and wear resistance, they are often more expensive and less ductile, which can pose challenges in applications requiring both precision and toughness. On the other hand, Al-SiC composites strike an optimal balance, offering both excellent wear resistance and enhanced damping characteristics, while still maintaining a relatively low density and good thermal conductivity. These advantages make Al-SiC particularly well-suited for the dynamic and thermal demands of ultraprecision machining systems.

## 2. Multi-Body Dynamics Analysis

### 2.1. Mathematical Theory

In actuator-based systems, the system responses can be classified into categories such as free response, un-tuned active damping, forced vibration, and adaptively tuned active damping. These categories describe the waveform of the system, taking into account the different types and properties of the actuators. In practical applications, however, over-tuning may present challenges, resulting in performance responses that depend more on inherent damping mechanisms rather than full tuning. Thus, introducing free response as an actuator into the system can improve its effectiveness and robustness. This study focuses on replacing heavier materials like steel with lighter alternatives, such as metal matrix composites (MMC), in multi-body dynamics simulations.

**Figure 1:**
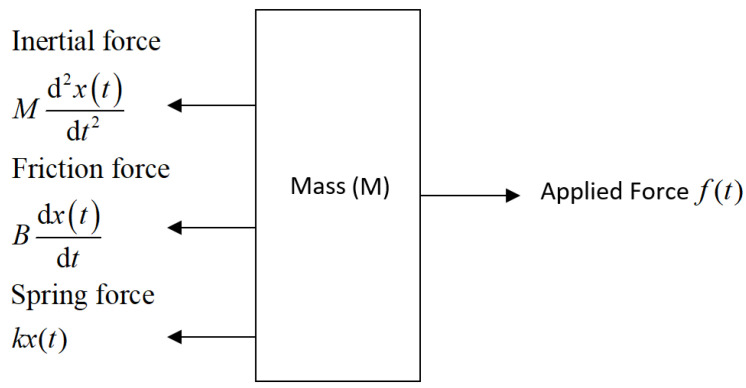
Free body diagram of the the forces acting on the slideway carriage.

From a mathematical perspective, the model can be simplified into a second-order differential equation, where the transfer function and Laplace transform are used to derive the system’s transient response under the assumption of zero initial conditions. This approach allows for the prediction of the system’s behaviour by analyzing the location of its poles. A second-order system can be represented by a canonical transfer function:

(1) $$\begin{array}{cc} G\left(s\right)=\frac{Y(s)}{U(s)}=\frac{K{\omega_{n}}^{2}}{s^{2}+2\xi \omega_{n}s+\omega_{n}} & \; \end{array}$$

where ξ denotes the damping ratio, $\omega_{n}$ represents the undamped natural frequency, and *K* indicates the DC gain or system constant. To simplify the system, we model the *Z*-axis as a spring-cart mechanism under unloaded conditions. This approach enables the determination of the stiffness response when the carrier is constructed using MMC material. Figure 1 presents the free body diagram of the system, with the key parameter constants specified as follows:

- *k* is the spring constant
- *M* is the mass of the *Z*-axis carriage
- *B* is the coefficient of viscous friction acting between the base and *Z*-axis carrier
- f(t) is applied force to the *Z*-axis
- x(t) is the displacement of the carriage from its equilibrium position
- $\dot{x}\left(t\right)=\frac{dx(t)}{dt}$ is the speed of *Z*-axis
- $\ddot{x}\left(t\right)=\frac{d^{2}x\left(t\right)}{dt^{2}}$ is the *Z*-axis acceleration

From the free body diagram, the acting forces can be calculated as follows:

(2) $$\begin{array}{cc} M\ddot{x}\left(t\right)+B\dot{x}\left(t\right)+kx\left(t\right)=f\left(t\right) & \; \end{array}$$

The Laplace transforming equation can then be found by the transfer function of the output X(s) to the input F(s) with zero initial condition: then,

(3) $$\begin{array}{c} \left(Ms^{2}+Bs+k\right)X\left(s\right)=F\left(s\right) \end{array}$$

(4) $$\begin{array}{cc} \frac{X(s)}{F(s)}=\frac{\frac{1}{M}}{s^{2}+\frac{B}{M}s+\frac{k}{M}}=\frac{k{\omega_{n}}^{2}}{s^{2}+2\xi \omega_{n}s+{\omega_{n}}^{2}} & \; \end{array}$$

From Equation (4), it is easy to understand that the spring constant k and the mass of the system *M* have direct influence on the undamped natural frequency $\omega_{n}$:

(5) $$\begin{array}{cc} \omega_{n}=\sqrt[{}]{\frac{k}{M}} & \; \end{array}$$

The viscous friction or damping coefficient B, as well as the other constants, influence the damping ratio ξ:

(6) $$\begin{array}{cc} \xi =\frac{B}{2\sqrt{kM}} & \; \end{array}$$

To demonstrate this concept, let us use the *Z*-axis as a practical example. In analyzing its time responses, we adhere to the theory for the *Z*-axis by assuming zero initial conditions, meaning the system begins from a state of rest. In this scenario, the transfer function of the system under zero initial conditions represents the zero-state response. However, it is also possible to assume the presence of internal energy stored in the initial condition, which would cause the system to exhibit a zero-input response. Mathematically, we model the *Z*-axis carriage as a spring-cart mechanism that is initially displaced from its equilibrium position and subsequently brought to rest by applying an external force. At time t = 0, the external force is suddenly removed, and the system undergoes vibrations until it comes to rest due to the potential energy stored in the spring.

Equations (7)–(12) describe the steps taken to simplify the model, starting with the differential equation and applying Laplace transformations that include the initial conditions. These equations specifically address the linear displacement factor of the system.

(7) $$\begin{array}{c} M(s^{2}X\left(s\right)-sx\left(0\right)-\frac{dx(0)}{dt}+B(sX\left(s\right)-x\left(0\right)+kX\left(s\right)=0 \end{array}$$

(8) $$\begin{array}{c} M\left(s^{2}X\left(s\right)-2.0s-0\right)+B\left(sX\left(s\right)-2.0\right)+kX\left(s\right)=0 \end{array}$$

(9) $$\begin{array}{c} M\left(s^{2}+Bs+k\right)X\left(s\right)-\left(2Ms+2B\right)=0 \end{array}$$

For simplification, the assumption below was created:

*B* = 0.1, *M* = 2, and *K* = 1, then the expression for X(s) can be found as follows:

(10) $$\begin{array}{c} X\left(s\right)=\frac{2Ms+2B}{Ms^{2}+Bs+k}=\frac{2s+2\frac{B}{M}}{s^{2}+\frac{B}{M}s+\frac{k}{M}}=\frac{2s+0.1}{s^{2}+0.05s+0.5} \end{array}$$

Using the transform pair, the displacement of the *Z*-axis can be described as follows:

(11) $$\begin{array}{c} \frac{s+k}{{\left(s+a\right)}^{2}+\omega^{2}}\to \frac{\sqrt{{\left(k-a\right)}^{2}+\omega^{2}}}{\omega }e^{-at}\sin\left(\omega t+\phi \right) \end{array}$$

where:

(12) $$\begin{array}{c} \phi =\arctan\frac{\omega }{\left(k-a\right)} \end{array}$$

### 2.2. Simscape Model Setup

**Figure 2:**
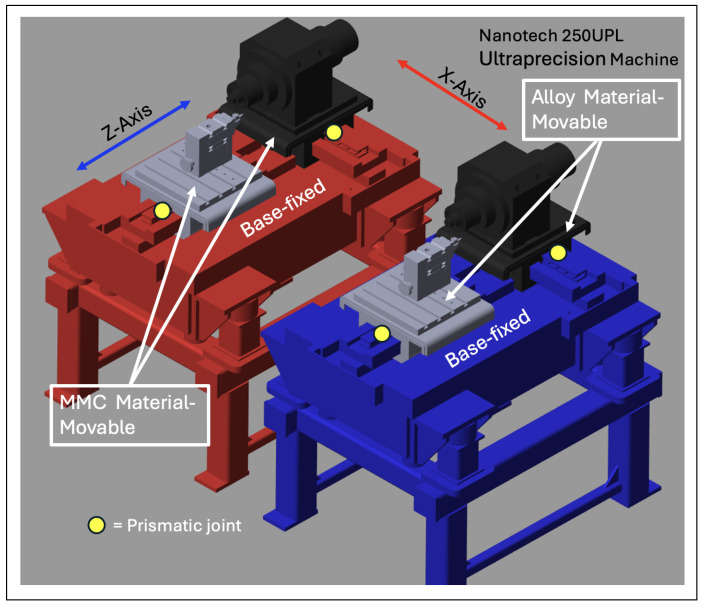
MBD Simscape model setup.

To conduct a multi-body dynamic (MBD) analysis of the ultraprecision diamond turning machine, a 3D CAD model of the slideways for both the *X* and *Z* axes is employed. This model allows for the examination of the dynamic effects and motion behaviour of the linear hydrostatic bearing component, particularly focusing on the carrier slide, when various materials are applied. In the analysis, the carrier, which makes up the entire moving part, is modeled as a single rigid body actuated by a mechanical spring, as outlined in the previous section. The programmable mass of the *Z*-axis is defined as follows:

- 80 kg (Alloy Steel)
- 27 kg (30% SiC, MMC)

and *X*-axis:

- 180 kg (Alloy Steel)
- 60 kg (30% SiC, MMC)

block system, the Simscape system block for the world reference, the solver, and the transformation configuration. The rigid body itself has interdependent system properties, such as geometric characteristics and inertia. Both the carrier and the base share the same set of properties, as shown in the carrier and base solid properties dialog box below. The only distinction between them lies in the core inertial properties. However, the solver and transformation configuration remain the same to maintain a consistent environmental sandbox setup. As shown in Figure 2, the MBD models are divided into two sections, each with varying the densities and masses for the MMC and alloy steel materials. The blue diamond turning machine represents the alloy steel material for both the *X* and *Z* axes, while the red diamond turning machine represents the MMC material.

The material properties of MMC (Al 2024) with an average particle size of 3 μm assigned to the hydrostatic bearing components. The material properties of MMC AL2024 are provided in Table 1 [13].

In the MBD simulations conducted using Simscape MATLAB-R2020b, the boundary conditions were defined by fixing the base of the slideway system, with prismatic joints enabling linear motion along the *X* and *Z* axes. Forces and displacements were applied as per the experimental setup. The simulations utilized a variable-step solver, which adjusts the time step for accuracy based on system dynamics. To ensure the reliability of the simulation results, the model was validated against available experimental data and benchmarked with simplified theoretical models, confirming that the simulated behaviour aligns with the expected performance.

Upon completing the modeling and configuration setup in the Simscape software MATLAB-R2020b, the MBD simulation model involved the application of a prismatic joint to both the *Z*-axis and *X*-axis. The prismatic joint allows for lateral displacement along a specific axis of motion. In this simulation, an initial displacement of 5 mm is assumed. The velocity is automatically calculated, taking into account the damping and stiffness of the mechanical input of the joint. Additionally, the intrinsic mechanics and self-actuation of the joint’s interaction between the two materials, alloy steel and MMC, are programmed into the prismatic joint. This programming includes setting a spring stiffness of 1000 N/m and a damping coefficient of 650 N/ms. The selected prismatic joint also includes kinematic sensing capabilities, enabling the automatic calculation or provision of position, velocity, and acceleration data from the control signal of the slideway control system.

The spring stiffness for the prismatic joint was determined methodologically as the overall spring stiffness (a constant) of the interconnected slideway body when subjected to an applied force, considering the maximum load-carrying capacity. At this point in the modeling process, the inclusion of the spring in the prismatic joint is based on an assumption, providing sufficient input data to predict and approximate the differentiation for the output response. This approach allows for a comparative analysis of the two materials under study. According to theoretical principles, the mechanical force required to accelerate the mass of the slideway’s structure and achieve a predefined final velocity is considered. For the sliding friction coefficient, it is ideally assumed to represent a free-friction system, where only a minimal applied force is needed to reach the desired motion values set in the programming. Although the mathematical model assumes a free-friction system for simplicity, it is acknowledged that in practical fluid hydrostatic bearings and air bearings, friction coefficients and thermal effects can significantly influence performance. In hydrostatic bearings, for instance, the load-carrying capacity and damping are highly dependent on fluid friction and temperature variations. For air bearings, the impact of friction is primarily related to the air-film characteristics and temperature changes, which can affect the stiffness and accuracy of the system. Future work will incorporate these factors to better evaluate their impact on system behaviour.

### 2.3. MBD Actuator

**Figure 3:**
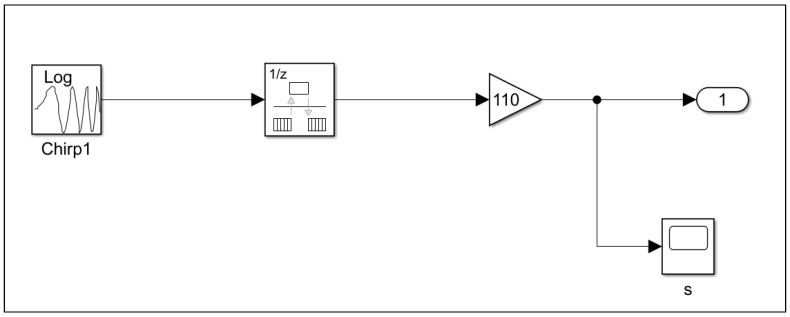
MBD Simscape actuator.

**Figure 4:**
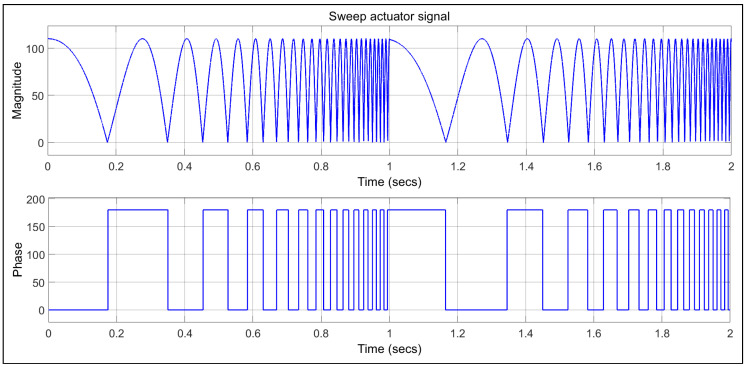
Actuator signal.

Subsequently, upon establishing the mechanical joints and initial conditions between the fixed and movable components of the linear hydrostatic bearing slide, an actuator is employed to induce motion in the motor. The actuator parameters listed in Table 2, and the setting for the MBD acctuatore presented in Figure 3 and Figure 4.

In the experiment, the actuator generates the output acceleration and force, which are then transmitted to the moving component of the interconnected body-carrier. It is hypothesized that the initial response of the actuator will result in a higher output value for the MMC material compared with the alloy steel carrier. Additionally, while both materials show damping ratios numerically below 1, it is expected that the actuator system’s damping ratio will be relatively higher for the MMC material.

Taking into account the mass and density of the materials used in this scenario, it is suggested that these properties are adequate to cause a noticeable change in the output. Furthermore, using lighter alternatives, such as the MMC material, allows the actuators to achieve a greater range of linear motion, despite the oscillatory behaviour typical of the second-order actuator system. In this case study, a sweep actuator model was utilized to explore the multi-phase motion characteristics of the linear motor. As shown in Figure 3 and Figure 4, a sweep signal covering a frequency range from 1 to 50 Hz, with a gain of 110 to amplify the magnitude to 5500 Hz, was generated. This signal was then applied to the prismatic joints of both the *X* and *Z* axes. The same parameters for the actuators and prismatic joints were used for both the alloy steel and MMC materials. The configuration parameters used to generate the actuator signals are outlined in Table 2. The actuator generates a signal within a frequency range of 1 to 50 Hz, beginning from phase zero and lasting for a sweep time of two seconds.

**Figure 5:**
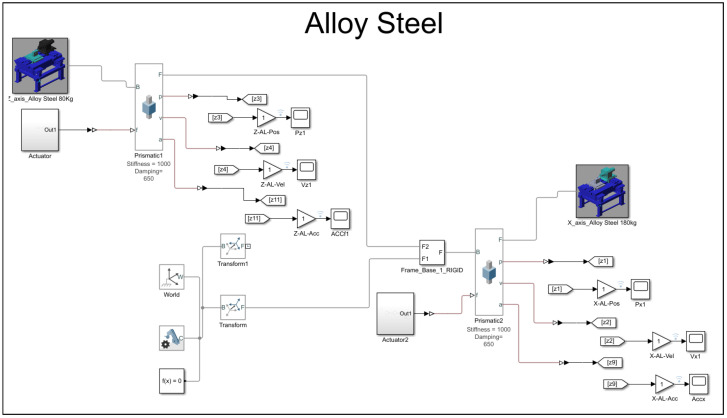
MBD, Simscap model setup for alloy material.

**Figure 6:**
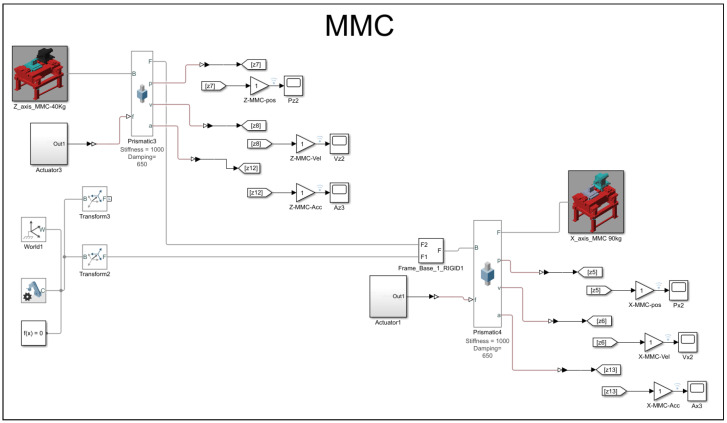
MBD, Simscap model setup for MMC material.

The sweep mode used in this study is computed in a logarithmic unidirectional fashion. Figure 5 and Figure 6 illustrate the multi-body dynamics models created with Simscape for the case study, specifically simulating the use of alloy steel and MMC materials, respectively.

### 2.4. MBD Discussion and Results

**Figure 7:**
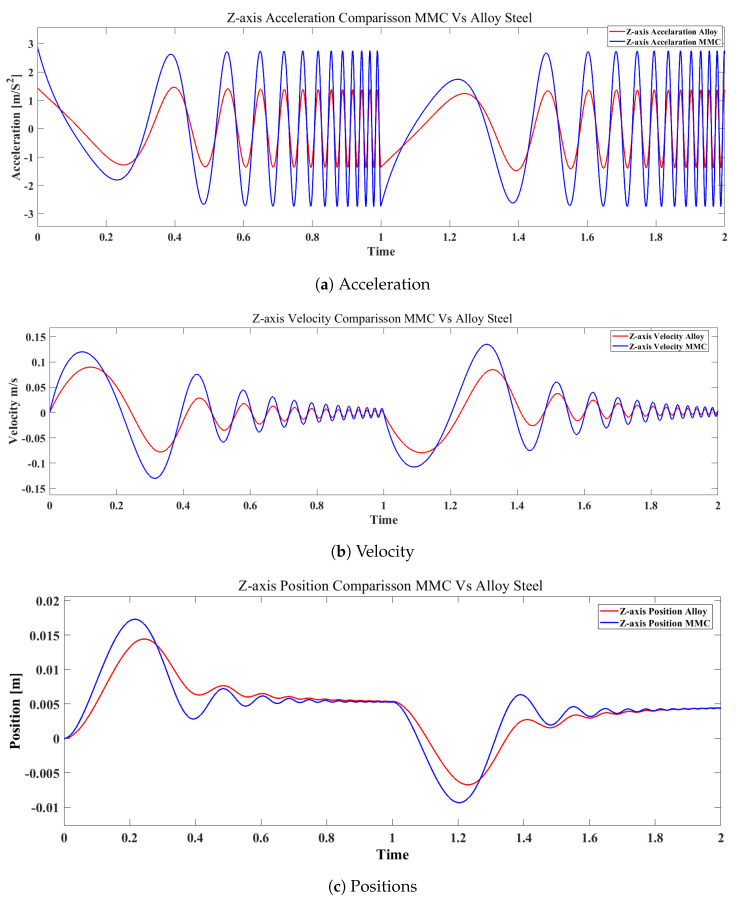
Simscape MBD result *Z*-axis: (**a**) *Z*-axis acceleration; (**b**) *Z*-axis velocity; (**c**) *Z*-axis position.

The obtained results include the position, velocity, and acceleration at the multi-body dynamics (MBD) scale. Initially, the approximate displacement, acceleration, and velocity were configured for the prismatic joints based on the specific actuation frequency and vibration of the *z*-axis and *x*-axis. As a result, the output data were calculated using approximate values to obtain the final comparison data for both the MMC and alloy steel materials. After completing the simulation, the output data for both the *X* and *Z*-axes using the alternative MMC material were plotted, and a comparison graph was created, as shown in Figure 7. Table 3 lists the output data, including approximate values for acceleration, velocity, and position. Analyzing the acceleration results reveals that the maximum value in the benchmark scenario for the MMC material exceeds that for alloy steel. This suggests that the lighter material, such as MMC, demonstrates a faster response at higher frequencies compared with the denser alloy steel. This finding supports the theory discussed earlier in the section on structural frequency response analysis. Upon reviewing the acceleration data in Table 3, it is evident that the maximum value is 1.46 m/s^2^ for the *Z*-axis with alloy steel, while for the MMC material, it reaches 2.8 m/s^2^.

The results show a substantial increase of over 50% in acceleration when the MMC material was used. This difference in values is particularly significant on the *X*-axis, where the mass is generally higher than that on the *Z*-axis. The calculated value for the *X*-axis with the MMC material is nearly equivalent to the *Z*-axis with the alloy steel material. This suggests that replacing heavier materials with lighter alternatives like MMC, as highlighted in the literature review, can lead to more efficient and effective designs, facilitating improved machining in areas such as hydrostatic bearings, ultraprecision machining, and precision engineering.

A further analysis of the velocity output data on the Z-axis confirms a significant improvement when using the MMC material compared with alloy steel. The maximum velocity recorded for alloy steel is 8.97 × 10^−2^ m/s, whereas it reaches 1.34 × 10^−1^ m/s for MMC. This indicates a notable increase in velocity when the MMC material is employed. An analysis of the positioning output data further supports the effectiveness of displacement when using the MMC material. The obtained output data, as shown in Table 3 and Figure 7c, display the response of the linear motor’s movement along its axis. It is clear that the MMC material demonstrates a more immediate and responsive behaviour compared with alloy steel. As illustrated in Figure 7c, the maximum linear displacement on the *Z*-axis is approximately 1.7 × 10^−2^ m (17 mm) for the MMC material and 1.4 × 10^−2^ m (14 mm) for alloy steel, both achieved within a time frame of 0.2 s. Analyzing these values highlights the benefits of using the MMC material in hydrostatic linear motors, particularly in precision engineering applications where the single-point diamond turning (SPDT) technique is used for freeform surface machining. This advantage effectively meets the demands of ultraprecision machining for freeform surfaces using the SPDT mode, offering a higher bandwidth frequency and extended stroke capability.

**Figure 8:**
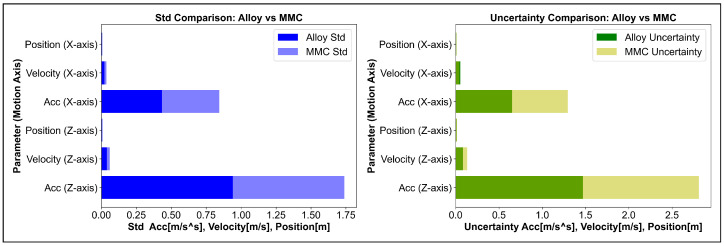
Statistic analysis of standard deviation and uncertainty.

Based on the results provided for both MMC and alloy materials, we can observe several key differences in the behaviour of each material when applied to the parameters of acceleration, velocity, and position. The standard deviation values for MMC, as shown in Figure 8, are significantly higher than those for alloy. For example, the standard deviation for acceleration on the *Z*-axis is 2.616 for MMC, compared with 1.364 for the alloy. This indicates that MMC experiences more variation in its response compared with the alloy, which might indicate a higher dynamic range or variability in the behaviour of the system when MMC is used. Further experimental work required to study the mechanical impacts of the MMC material for evaluating and identifying those variables. The standard deviation of the alloy is lower, indicating a more consistent performance. Considering the uncertainty, MMC also exhibits higher uncertainty values compared with the alloy. For example, the uncertainty in acceleration for the *Z*-axis is 2.810 for MMC, compared with 1.470 for the alloy. This suggests that MMC may have more variability in its behaviour, which might be linked to its lighter weight and possibly less predictable damping characteristics compared with the alloy.

**Key Differences:**

- **Higher Dynamic Performance for MMC:** The MMC material demonstrates a higher maximum acceleration, velocity, and displacement compared to the alloy, making it more responsive and better suited for high-performance systems requiring quick motion, such as ultraprecision machining.
- **Increased Variation in MMC:** The higher standard deviation and uncertainty for MMC suggest that, while it can achieve a better performance, its behavior might be less predictable or more variable compared with the alloy. This could be a result of the specific material composition or the inherent properties of MMCs.
- **Alloy Stability:** The alloy, on the other hand, provides more stable and consistent results with a lower variation in performance, which could be beneficial in situations where reliability and predictability are more important than raw performance.

## 3. Conclusions

This study compares the performance of metal matrix composite (MMC) and alloy steel materials in the context of linear slideways for ultraprecision machining. Multi-body dynamic (MBD) simulations were conducted to evaluate the dynamic behaviour of the two materials, considering key performance parameters such as acceleration, velocity, and displacement.

The results demonstrate that the MMC material significantly outperforms alloy steel in terms of acceleration, velocity, and displacement. Specifically, MMC exhibited higher acceleration and velocity values, suggesting that it provides a faster response and improved dynamic performance compared with alloy steel. Additionally, MMC showed a longer linear displacement stroke, offering advantages for ultraprecision machining, particularly in applications utilizing slow tool servo (STS) techniques.

These finding indicate that MMC materials, with their superior dynamic characteristics, are a promising alternative to traditional alloy steel for improving the performance of linear slideways in ultraprecision machining applications. The study highlights the potential benefits of using lighter materials like MMC to enhance the overall efficiency and capability of precision engineering systems. Nevertheless, the higher standard deviation and uncertainty observed in the MMC material are expected due to its lighter nature and more dynamic response, highlighting the need for additional experimental work to better understand the mechanical impacts and material strength of MMC in practical applications.

Furthermore, this study contributes to advancing precision engineering and the development of linear slideways such as airbearing and hydrostatic bearing. The application of MMC opens new possibilities for achieving a better performance and more efficient machining. Further research could lead to the development of linear slideways that overcome load-carrying capacity and bandwidth limitations, enhancing the capabilities of ultraprecision machines in freeform surface machining. The conclusions drawn in this study are primarily based on simulation results. Experimental work is currently underway and will be presented in future research to further validate and enhance the reliability of these findings.

## Figures and Tables

**Table 1:** Material properties of the AL-MMC (Al 2024).

Al-MMC (Average Particle Size: 3 μm, 30% SiC)	
Density ρ (g/cm^3^)	2.9
Youngs modulus E (GPa)	115
Hardness (HV 0.1)	276
Thermal conductivity κ (W/(mK))	145
Specific heat capacity c (J/(kgK))	828
Thermal diffusivity a (m^2^/s)	6.04 × 10^−5^
Coefficient of thermal expansion α (1/K)	15.6 × 10^−6^
Residual (compressive) stress prior to turning (MPa)	−4

**Table 2:** MBD actuator parameters.

Actoator Parameters							
Frequency Sweep	Sweep Mode	Initial Frequency [Hz]	Target Frequency [Hz]	Target Time [s]	Sweep Time [s]	Initial Phase [rad]	Sample Time
Logarithmic	Unidirectional	1	50	1	1	0	1/8000

**Table 3:** MBD signal statistic comparison, MMC vs. alloy steel.

			Signal Statistic					
			Max	Min	Peak	Mean	Meadian	RMS
**Alloy**	***Z*** **-axis**	**Acceleration [m/s^2^]**	1.46	−1.48	2.9	1.30 × 10^−3^	7.39 × 10^−3^	9.40 × 10^−1^
		**Velocity [m/s]**	8.97 × 10^−2^	−7.95 × 10^−2^	1.69 × 10^−1^	2.20 × 10^−3^	7.60 × 10^−4^	3.96 × 10^−2^
		**Position [m]**	1.40 × 10^−2^	−6.73 × 10^−3^	2.11 × 10^−2^	4.29 × 10^−3^	4.30 × 10^−3^	6.10 × 10^−3^
	***X*** **-axis**	**Acceleration [m/s^2^]**	6.47 × 10^−1^	−6.55 × 10^−1^	1.3	2.30 × 10^−3^	4.90 × 10^−3^	4.33 × 10^−1^
		**Velocity [m/s]**	5.50 × 10^−2^	−5.00 × 10^−2^	1.05 × 10^−1^	1.80 × 10^−3^	1.47 × 10^−3^	2.18 × 10^−2^
		**Position [m]**	9.80 × 10^−3^	−2.23 × 10^−3^	1.20 × 10^−2^	4.25 × 10^−3^	3.56 × 10^−3^	5.44 × 10^−3^
**MMC**	***Z*** **-axis**	**Acceleration [m/s^2^]**	2.87	−2.75	5.62	2.00 × 10^−3^	8.03 × 10^−3^	1.74 × 10^0^
		**Velocity [m/s]**	1.34 × 10^−1^	−1.30 × 10^−1^	2.64 × 10^−1^	2.18 × 10^−3^	9.20 × 10^−4^	5.90 × 10^−2^
		**Position [m]**	1.70 × 10^−2^	−9.33 × 10^−3^	2.60 × 10^−2^	4.30 × 10^−3^	4.40 × 10^−3^	6.70 × 10^−3^
	***X*** **-axis**	**Acceleration [m/s^2^]**	1.29	−1.3	2.6	1.40 × 10^−3^	6.90 × 10^−3^	8.44 × 10^−1^
		**Velocity [m/s]**	8.44 × 10^−2^	−7.70 × 10^−4^	1.59 × 10^−1^	2.20 × 10^−3^	7.70 × 10^−4^	3.65 × 10^−2^
		**Position [m]**	1.38 × 10^−2^	−6.17 × 10^−3^	1.90 × 10^−2^	4.28 × 10^−3^	4.34 × 10^−3^	6.02 × 10^−3^

## Data Availability

The data are unavailable due to privacy and ethical restrictions.
